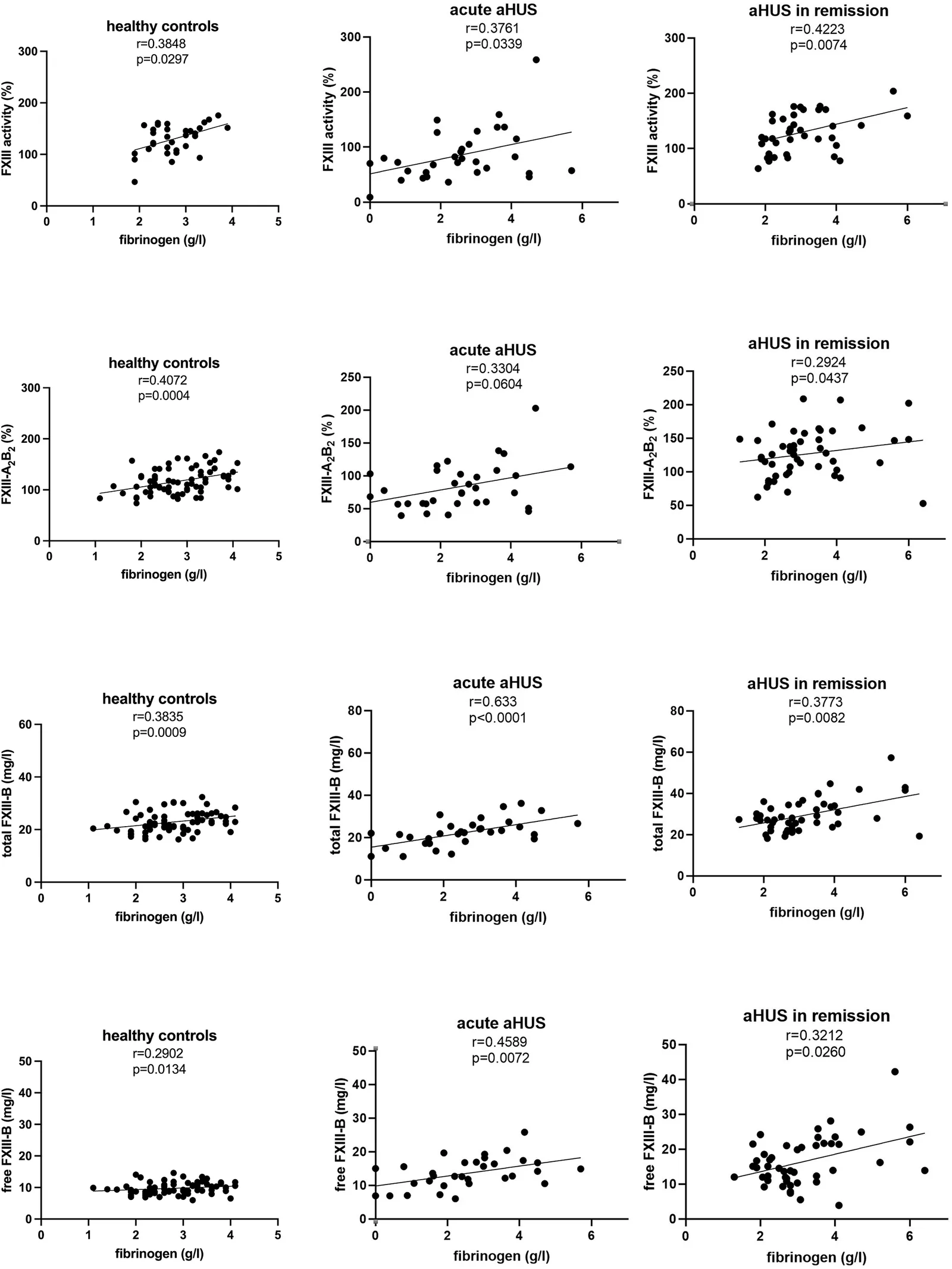# Beyond complement: comprehensive assessment of factor XIII plasma dynamics and genetics in atypical hemolytic uremic syndrome

**DOI:** 10.1016/j.rpth.2026.106891

**Published:** 2026-07-28

**Authors:** Noémi Janszky, Éva Katona, György Sinkovits, Dorottya Csuka, Ágnes Haris, Velibor Tasic, Nora Abazi-Emini, Christof Aigner, Alice Schmidt, Gere Sunder-Plassmann, Silvie Rajnochová Bloudíčková, Zoltán Prohászka, László Muszbek, Ágnes Szilágyi

**Affiliations:** 1 Research Laboratory, Department of Internal Medicine and Haematology, Semmelweis University, Budapest, Hungary; 2 Division of Clinical Laboratory Science, Department of Laboratory Medicine, Faculty of Medicine, University of Debrecen, Debrecen, Hungary; 3 Department of Internal Medicine and Nephrology, Budapesti Péterfy Hospital-Outpatient Clinic, Budapest, Hungary; 4 Medical Faculty Skopje, University Children’s Hospital, Skopje, North Macedonia; 5 Divison of Nephrology and Dialysis, Department of Medicine III, Medical University Vienna, Vienna, Austria; 6 Department of Nephrology, Transplant Center, Institute for Clinical and Experimental Medicine, Prague, Czech Republic; 7 Department of Internal Medicine and Haematology, Research Group of Immunology and Hematology, Hungarian Research Network - Semmelweis University (HUN-REN-SU), Semmelweis University, Budapest, Hungary

**Keywords:** atypical haemolytic uraemic syndrome, blood coagulation factors, factor XIII, FXIII-B subunit, genetic variation

## Abstract

**Background:**

Atypical hemolytic uremic syndrome (aHUS) is a distinct form of thrombotic microangiopathy characterized by formation of fibrin-rich thrombi. Factor XIII (FXIII), a key fibrin-stabilizing enzyme, may therefore represent an unexplored contributor to the pathophysiology of aHUS, and a comprehensive evaluation of its plasma dynamics and genetic background is lacking.

**Objectives:**

This study aimed to evaluate plasma FXIII levels and *F13A1/F13B* genetic variants in patients with aHUS.

**Methods:**

The cohort comprised 102 patients with aHUS and 73 healthy controls. FXIII activity was measured by ammonia release assay, FXIII antigen levels by ELISA, and *F13A1/F13B* variants by high-resolution melting analysis with confirmatory Sanger sequencing.

**Results:**

FXIII activity was decreased in aHUS patients during the acute phase (median 72.8%; IQR, 52.8%-121.0%) versus those in remission (123.1%; IQR, 97.9%-156.3%; *P* < .0001) and healthy controls (136.4%; IQR, 108.5%-151.5%; *P* < .0001). FXIII-A_2_B_2_ levels paralleled FXIII activity. Free FXIII-B level was elevated in patients in remission (14.7 mg/L; IQR, 11.9-21.2) versus those in acute phase (12.7 mg/L; IQR, 10.1-16.6; *P* = .02) and healthy controls (9.4 mg/L; IQR, 8.7-10.9; *P* < .0001). Genetic analysis identified 6 common and 4 rare nonsynonymous *F13A1/F13B* variants, with allele and genotype frequencies comparable to European individuals in the 1000Genomes Project. Apart from *F13B* variant Tyr100Ter, which was associated with reduced FXIII-B, the rare variants did not show a clear impact on FXIII plasma levels.

**Conclusion:**

This comprehensive genetic and plasma study of FXIII in aHUS argues against it having a causal role, while the remission-associated increase in free FXIII-B supports it as a promising biomarker candidate for this rare but severe disease.

## Essentials

• The role of factor XIII (FXIII) in atypical hemolytic uremic syndrome (aHUS) remains unclear.
• FXIII plasma levels and related genes were analyzed in people diagnosed with aHUS.
• FXIII-A was lower during the acute phase, and FXIII-B was higher when patients recovered.
• Genetic changes appeared to have little impact, but evaluation of the FXIII-B subunit as a marker of disease activity may be warranted.

## Graphical abstract

**Figure:**
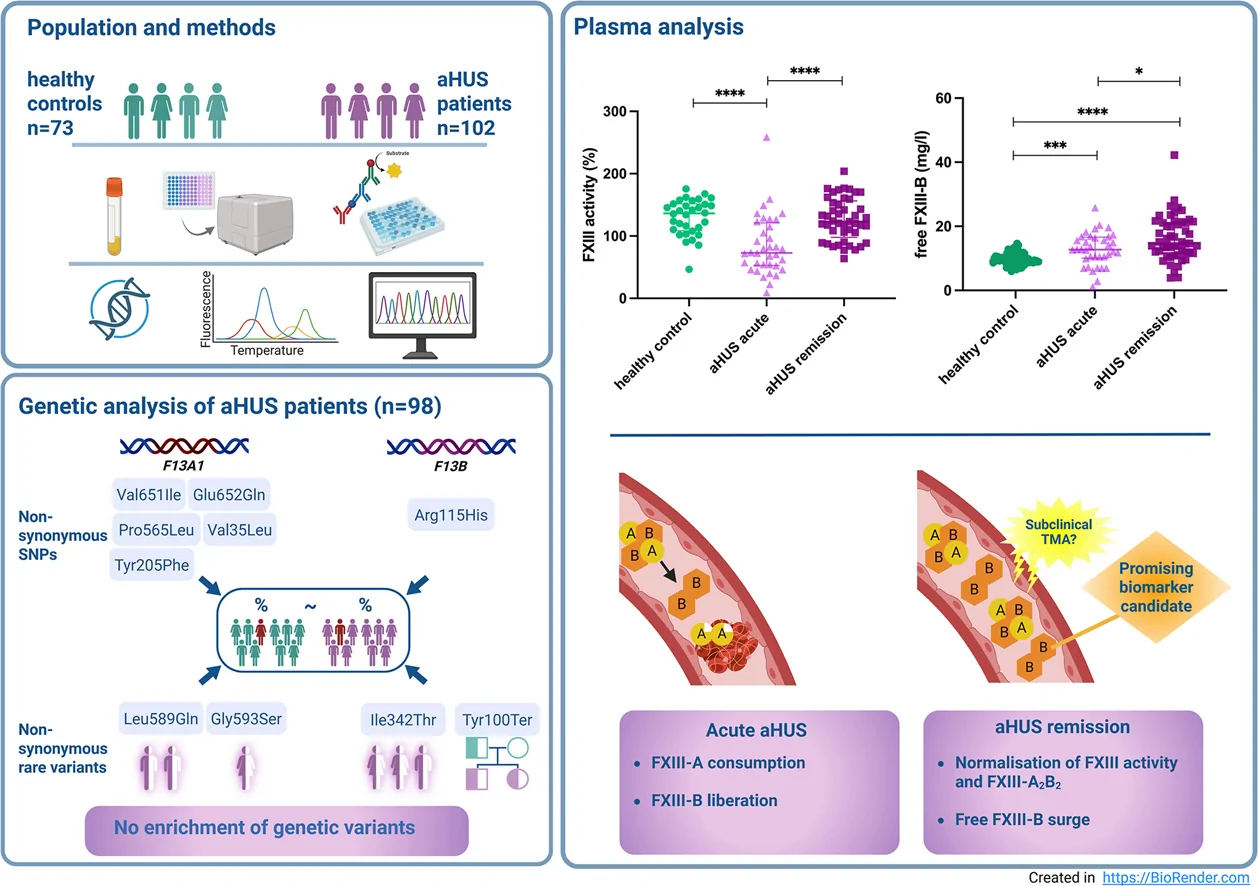

## 1 Introduction

Atypical hemolytic uremic syndrome (aHUS) is a distinct clinical entity characterized by microangiopathic hemolytic anemia, thrombocytopenia, and renal impairment. Of particular interest, the thrombotic microangiopathy (TMA) in aHUS results from dysregulated activation of the complement system on endothelial cell surfaces, leading to sustained endothelial injury and promoting microvascular thrombosis, especially within the renal circulation [1]. In keeping with current clinical practice and contemporary expert discussions on disease classification, we use the traditional term aHUS in this manuscript, while acknowledging recent proposals for mechanism-based terminology such as complement-mediated TMA [2,3].

Rare variants and polymorphisms in genes encoding the complement system’s regulatory proteins such as factor H (*CFH*) and factor I (*CFI*), membrane cofactor protein (*CD46*), as well as the complement components C3 (*C3*) and factor B (*CFB*) represent risk factors for aHUS [4]. Although a substantial proportion of complement gene alterations have been functionally characterized and linked to the development of aHUS, pathogenic variants in these genes are undetectable in approximately 30% to 40% of aHUS cases. Furthermore, familial aHUS often presents with incomplete penetrance, and the aforementioned facts point to unexplored factors in the development of aHUS [4].

In aHUS, the main histopathological feature is the appearance of fibrin microthrombi in the microvasculature, and the interaction between the complement and coagulation cascades is becoming increasingly recognized. These facts rationalize the investigation of genes encoding proteins of the coagulation and fibrinolytic system [5]. Aside from pathogenic variants in *DGKE*, which define a distinct, complement-independent form of aHUS [6], rare variants in several coagulation pathway genes have been investigated for a potential contributory role in the pathophysiology of aHUS. In addition to their well-established roles in the coagulation and fibrinolytic systems, thrombomodulin and vitronectin have recently been implicated in the regulation of the complement system, and the contributions of rare variants in thrombomodulin (*THBD*) [7] and vitronectin (*VTN*) [8] in aHUS are still under active investigation.

Furthermore, pathogenic variants in the plasminogen gene (*PLG*) have also been associated with aHUS [9]. Such alterations may impair plasmin generation, resulting in inadequate fibrin thrombus degradation. The ensuing persistence of microvascular thrombi contributes to the TMA characteristic of aHUS. Recent studies further suggest that TMA associated with pathogenic *PLG* variants may represent a distinct clinicopathological subgroup with specific therapeutic requirements, highlighting the need for more comprehensive characterization of the underlying mechanisms [10].

Building on these observations, our attention turned to additional hemostatic factors that might modulate microvascular injury. In this context, another coagulation factor, factor XIII (FXIII), plays a major role in fibrin clot stability; FXIII is crucial for fibrin cross-linking and for incorporation of antifibrinolytic proteins such as α2-antiplasmin into the fibrin network, thereby stabilizing thrombi against plasmin-mediated lysis [11]. Apart from a single study assessing a specific FXIII single nucleotide polymorphism (SNP) in a mixed TMA population [12], the association between FXIII and the pathophysiology of aHUS has not been characterized, and a comprehensive evaluation of FXIII in aHUS has, to our knowledge, not previously been undertaken.

FXIII circulates in the plasma in the form of a heterotetramer complex (FXIII-A_2_B_2_) consisting of 2 catalytic A subunits (FXIII-A) and 2 transport and regulatory B subunits (FXIII-B). The FXIII-B subunit circulates in an approximately 2-fold molar excess relative to the FXIII-A subunit [13] and primarily functions as a carrier molecule that stabilizes FXIII-A and prolongs its plasma half-life. When FXIII-A subunits are activated, the B subunits dissociate from the FXIII-A_2_B_2_ complex and remain in the circulation as free FXIII-B, which predominantly circulates bound to fibrinogen [14]. The characteristic presence of fibrin thrombi in aHUS, together with the key role of FXIII in fibrin clot stabilization, raises the possibility that FXIII may modulate thrombus formation and persistence in this disorder.

Therefore, the main goal of this work was to perform a comprehensive functional and genetic analysis of FXIII in patients with aHUS, a rare but severe TMA, for which such data have been lacking. More specifically, we aimed to 1) measure plasma FXIII activity and antigen levels in an aHUS cohort including patients in the early, acute phase and patients in hematologic remission, and 2) investigate the potential genetic background of the detected FXIII alterations through systematic *F13A1/F13B* variant characterization.

## 2 Methods

### 2.1 Patients and controls

In our study, 102 unrelated patients diagnosed with aHUS and 73 healthy controls were included. In addition, the family of 1 aHUS patient (1 affected sibling and the 2 unaffected parents) was screened. All participants had been previously registered in our laboratory for diagnostic evaluation between 2009 and 2018 and originated from Hungary or from the following European countries: Austria, Bosnia and Herzegovina, the Czech Republic, Lithuania, North Macedonia, Serbia, Slovakia, Slovenia, and Ukraine. Biological samples were stored at −80 °C until analysis. Of these, samples from 98 patients were analyzed for *F13A1* and *F13B* variants, and plasma measurements were performed in 89 aHUS patients, 36 of whom were sampled during the acute phase before plasma therapy initiation. Samples from patients in remission were sent to our laboratory for routine follow-up, family screening, or genetic evaluation prior to kidney transplantation. The criteria for remission were defined as a platelet count of >150 × 10^9^/L and the absence of hemolysis, regardless of kidney function. The acute phase and remission groups consisted of different individuals, except for 4 patients who had both acute phase and remission samples. In these latter cases, the remission samples were collected ≥1 year after the acute episode during follow-up visits.

As a control group, we included samples from healthy, unrelated Caucasian individuals who had been examined as part of routine occupational health assessments at Semmelweis University. Apart from isolated cases of hypertension, nephrolithiasis, cholecystolithiasis, and hyperlipidemia, no systemic morbidities were reported at the time of blood sampling.

The study protocol was approved by the Institutional Review Board of Semmelweis University and the Hungarian National Ethics Committee (ETT-TUKEB; approval no. 8361-1/2011-EKU and IV/4403-2/2020/EKU), and written informed consent was obtained from all participants or their legal guardians prior to enrollment. The research was conducted in accordance with the ethical principles of the Declaration of Helsinki and complied with all applicable local and national regulatory requirements.

The clinical diagnosis of HUS was established in the presence of nonimmune hemolytic anemia, acute thrombocytopenia, and acute kidney injury. Furthermore, aHUS was diagnosed on an exclusion basis. Accordingly, HUS patients were considered to have aHUS if neither of the following clinical situations occurred: (1) HUS appeared after enterohaemorrhagic *Escherichia coli* gastroenteritis, severe purulent pneumonia, or meningitis, or (2) HUS was associated with an obvious comorbidity. In addition, age younger than 6 months or older than 5 years, delayed onset, HUS relapses, previously suspected HUS, unexplained anemia or thrombocytopenia, and asynchronous HUS in the family were also suggestive of aHUS. We excluded patients with anti-factor H antibody–associated aHUS, as this autoimmune subtype has a distinct complement genetic risk profile and would constitute a separate subgroup. The characteristics of the included aHUS patients and healthy controls are detailed in Table 1.

**Table 1:** Characteristics of aHUS patients and healthy controls.

Characteristic	Healthy controls (*n* = 73)	aHUS patients (*n* = 102)
Male sex, *n* (%)	26 (36%)	41 (40%)
Age at sampling, y, mean ± SD	38.4 ± 10.3	23.9 ± 18.2^a^
Clinical manifestations, *n* (%)		
More than 1 aHUS episode	—	28 (27%)
Progression to ESKD	—	36 (35%)
aHUS after respiratory tract infection	—	5 (5%)
aHUS after gastroenteritis	—	9 (9%)
aHUS episode before age 1 y	—	12 (12%)
Postpartum aHUS	—	12 (12%)
Neurological symptoms	—	5 (5%)
ESKD of unknown origin, aHUS after kidney transplantation	—	7 (7%)
Relapse(s) after kidney transplantation	—	16 (16%)
Familial aHUS	—	10 (10%)

Laboratory parameters, median (IQR)	Healthy controls	aHUS remission	aHUS acute
Complement C3, g/L	1.2 (1.1-1.4) (*n* = 73)	0.98 (0.8-1.3) (*n* = 56)	0.8 (0.6-1.1) (*n* = 46)
D-dimer, mg/L	0.3 (0.2-0.5) (*n* = 62)	0.46 (0.3-0.7) (*n* = 47)	3.4 (1-5.2) *(n* = 32)
Fibrinogen, g/L	2.8 (2.3-3.3) (*n* = 72)	2.8 (2.2-3.8) (*n* = 48)	2.6 (1.6-3.6) (*n* = 33)
Pathogenic/likely pathogenic variants in aHUS-associated genes, *n* (%)	—	37 (36%)	

aHUS, atypical hemolytic uremic syndrome; ESKD, end-stage kidney disease.

a aHUS patients are significantly younger than the healthy controls (*P* < .0001).

During the diagnostic evaluation of all included patients (*n* = 102), the activity of the alternative pathway, levels of complement factors B, H, and I, as well as C3 were determined, as published previously [15]. Diagnostic workup of patients also included the sequencing of the whole coding regions of the genes encoding complement factor H (*CFH*), factor I (*CFI*), factor B (*CFB*), C3 (*C3*), membrane cofactor protein (*CD46*), and thrombomodulin (*THBD*).

### 2.2 Plasma measurements

Plasma FXIII-A_2_B_2_, total FXIII-B, and free FXIII-B levels were measured in the samples from aHUS patients during the acute phase and in hematologic remission according to previously published protocols [13,16,17].

Activity of FXIII in both aHUS patients and healthy controls was measured in citrated plasma using a commercially available kit (TECHNOCHROM FXIII Kit; Technoclone GmbH). Because of the limited volume of stored plasma, FXIII activity was determined in 36 aHUS patients in the acute phase, 45 in remission, and 32 healthy controls.

We also analyzed plasma fibrinogen and D-dimer concentrations in aHUS patients, as well as in healthy controls, using the same samples that were assessed for FXIII plasma levels. Plasma fibrinogen levels were quantified using the Clauss method (Dia-FIB; Diagon), while D-dimer concentrations were determined through immunoturbidimetry (Dia-D-DIMER; Diagon).

### 2.3 Molecular genetic analysis

Genetic analysis of 98 aHUS patients was performed to screen for variants in the genes encoding the A and B subunits of FXIII (*F13A1* and *F13B*). Genomic DNA was extracted from mononuclear cells of EDTA-anticoagulated blood using the salting out technique [18].

### 2.4 Primer design

Using Primer Premier software (Premier Biosoft International), 2 pairs of forward and reverse primers were designed for the 15 exons of *F13A1* and the 12 exons of *F13B*. The outer primer pair was used for sequencing and the primer pair closer to the exon was used for melting point analysis. Primer sequences are available upon request.

### 2.5 HRM analysis

For genetic screening of the *F13A1* and *F13B* genes, high-resolution melting (HRM) analysis was performed using a Rotor-Gene Q (QIAGEN) real-time PCR device. PCR conditions for HRM were optimized individually for each exon to ensure maximal accuracy. Amplification of *F13A1* exon 1 was performed with AccuMelt HRM SuperMix (Quantabio), whereas *F13A1* exons 2-14 and *F13B* exons 1 and 4–12 were analyzed using MeltDoctor HRM Master Mix containing SYBR Green dye (Thermo Fisher Scientific). Each PCR reaction was carried out in a total volume of 20 μL, comprising approximately 50 ng DNA sample and either 10 μL 1× AccuMelt HRM SuperMix with 0.4 μM forward and reverse primers or 10 μL 1× MeltDoctor HRM Master Mix with 0.3 μM forward and reverse primers. Thermocycling started with a 10-minute long denaturation step at 95 °C, followed by 40 amplification cycles. Each cycle included denaturation at 95 °C, while annealing and extension conditions varied between exons to achieve optimal amplification; detailed parameters are available upon request. During the melting point analysis (melt) phase, the temperature of the sample was increased from 62 °C to 90 °C by 0.1 °C every 2 seconds, while the decrease in fluorescence was continuously detected. Samples of known genotype were used as controls for the measurements.

### 2.6 DNA sequencing

For validation and identification of the detected variants, Sanger sequencing applying BigDye chemistry was used following PCR amplification with GoTaq DNA Polymerase (Promega). PCR conditions are available upon request. Genotyping by HRM was not clear in case of 3 exons (exon 15 of *F13A1* and exons 2 and 3 of *F13B*), these regions were sequenced in all samples [19].

### 2.7 Statistical analysis

The nonparametric Mann–Whitney U-test was used to compare 2 independent groups, and data were described using the median and IQR. To determine correlations between plasma parameters, Spearman’s rank correlation test was applied. The chi-squared test was used to compare allele and genotype frequencies of our aHUS patient group to the healthy European population of 1000Genomes Project.

To analyze the representation of rare variants among aHUS patients compared with healthy individuals from the 1000Genomes Project, the collapsing method was used according to Li et al. [20].

All statistical analyses were performed with GraphPad Prism 8 (GraphPad Software), and *P* < .05 was considered statistically significant.

## 3 Results

### 3.1 Plasma measurements

**Figure 1:**
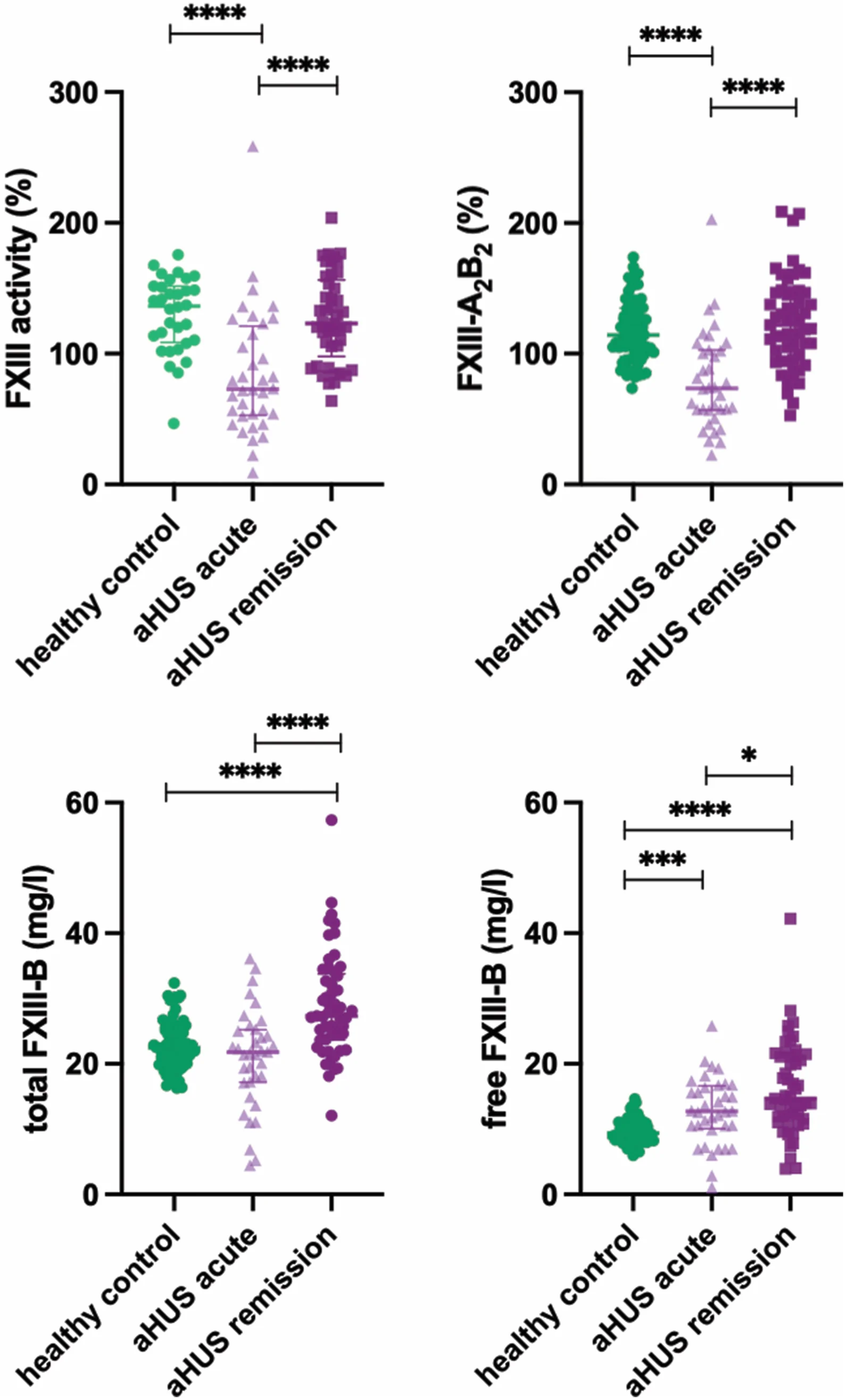
Factor III (FXIII) activity and antigen levels in the plasma of patients with atypical hemolytic uremic syndrome (aHUS) and healthy controls. ∗*P* < .05; ∗∗*P* < .01; ∗∗∗*P* < .001; ∗∗∗∗*P* < .0001.

The results of plasma FXIII measurements are summarized in Table 2 and depicted in Figure 1. Decreased FXIII activity and FXIII-A_2_B_2_ levels were detected in patients with aHUS during the acute phase compared with both aHUS patients in remission and healthy controls. Conversely, elevated concentrations of total and free FXIII-B subunits were observed in aHUS patients in remission compared with both patients in the acute phase and healthy individuals. Notably, the free portion of FXIII-B was elevated in patients with aHUS not only in remission but also during acute episode compared with healthy individuals.

**Table 2:** FXIII activity and FXIII antigen plasma levels.

Activity/level	Healthy controls	Acute aHUS	aHUS remission	*P* value (control vs acute)	*P* valuecontrol vs remission)	*P* value (acute vs remission)
FXIII activity, %	136.4 [108.5-151.5] (*n* = 32)	72.8 [52.8-121] (*n* = 36)	123.1 [97.9-156.3] (*n* = 45)	<.0001	.56	<.0001
FXIII-A_2_B_2_, %	114.3 [97.9–134.9] (*n* = 73)	73.7 [59.9-102.8] (*n* = 36)	121.8 [97.4-147.1] (*n* = 53)	<.0001	.23	<.0001
Total FXIII-B, mg/L	22.5 [20-25.5] (*n* = 73)	21.8 [17.2-25.2] (*n* = 36)	27.3 [23.7-33.8] (*n* = 53)	.20	<.0001	<.0001
Free FXIII-B, mg/L	9.4 [8.7-10-9] (*n* = 73)	12.7 [10.1-16.6] (*n* = 36)	14.7 [11.8-21.2] (*n* = 53)	.0002	<.0001	.02

Continuous variables are expressed as median [IQR].

aHUS, atypical hemolytic uremic syndrome; FXIII, factor XIII.

Plasma fibrinogen levels in aHUS patients remained within the reference range, with no significant differences between patients in remission and those in the acute phase or compared with healthy controls (Table 1). Fibrinogen showed positive correlations with FXIII parameters (FXIII activity, FXIII-A_2_B_2_, and total and free FXIII-B) with varying strengths across healthy controls, patients with acute aHUS, and patients in remission. Notably, in acute aHUS, the correlation with FXIII-A_2_B_2_ did not reach statistical significance, although a clear trend was observed. Corresponding *r* and *P* values and graphical representations are provided in the Supplementary Figure.

In contrast, D-dimer concentrations were elevated in both aHUS groups compared with healthy controls (remission vs controls: *P* = .02; acute vs controls: *P* < .0001), and were significantly higher during the acute phase than in remission (*P* < .0001) (Table 1).

### 3.2 Genetic screening

#### 3.2.1 Identification of nonsynonymous SNPs

During the genetic screening of aHUS patients, we found altogether 6 nonsynonymous SNPs in the *F13A1* and *F13B* genes: Val35Leu (rs5985), Tyr205Phe (rs3024477), Pro565Leu (rs5982), Val651Ile (rs5987), and Glu652Gln (rs5988) in *F13A1*, and Arg115His (rs6003) in *F13B*.

**Figure 2:**
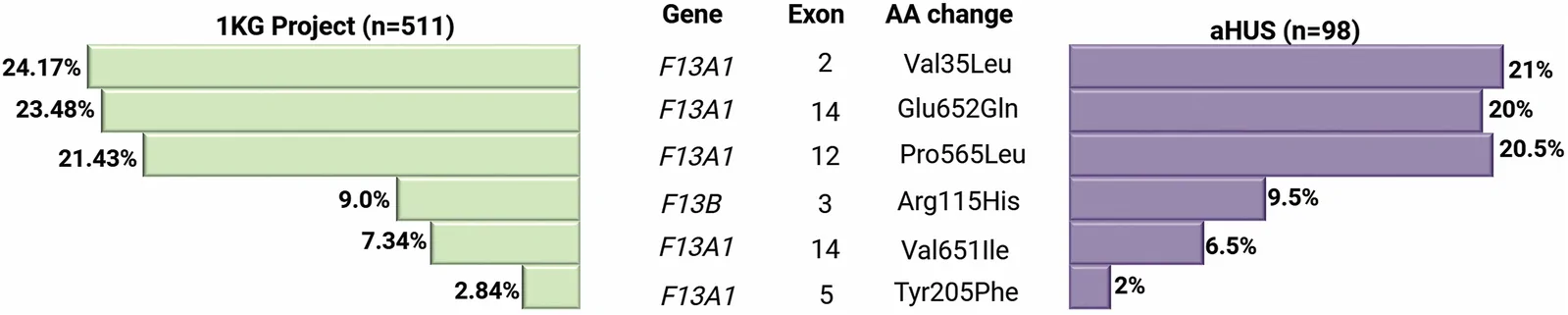
Summary of *F13A1* and *F13B* nonsynonymous single nucleotide polymorphisms and their minor allele frequencies in our aHUS cohort and in the European population of the 1000Genomes (1KG) Project. AA, amino acid; aHUS, atypical hemolytic uremic syndrome. Created in BioRender.com.

No significant difference was found when the allele and genotype frequencies of these common variants were compared with those reported in the European healthy population from the 1000Genomes Project (Figure 2).

#### 3.2.2 Identification of nonsynonymous rare variants

**Figure 3:**
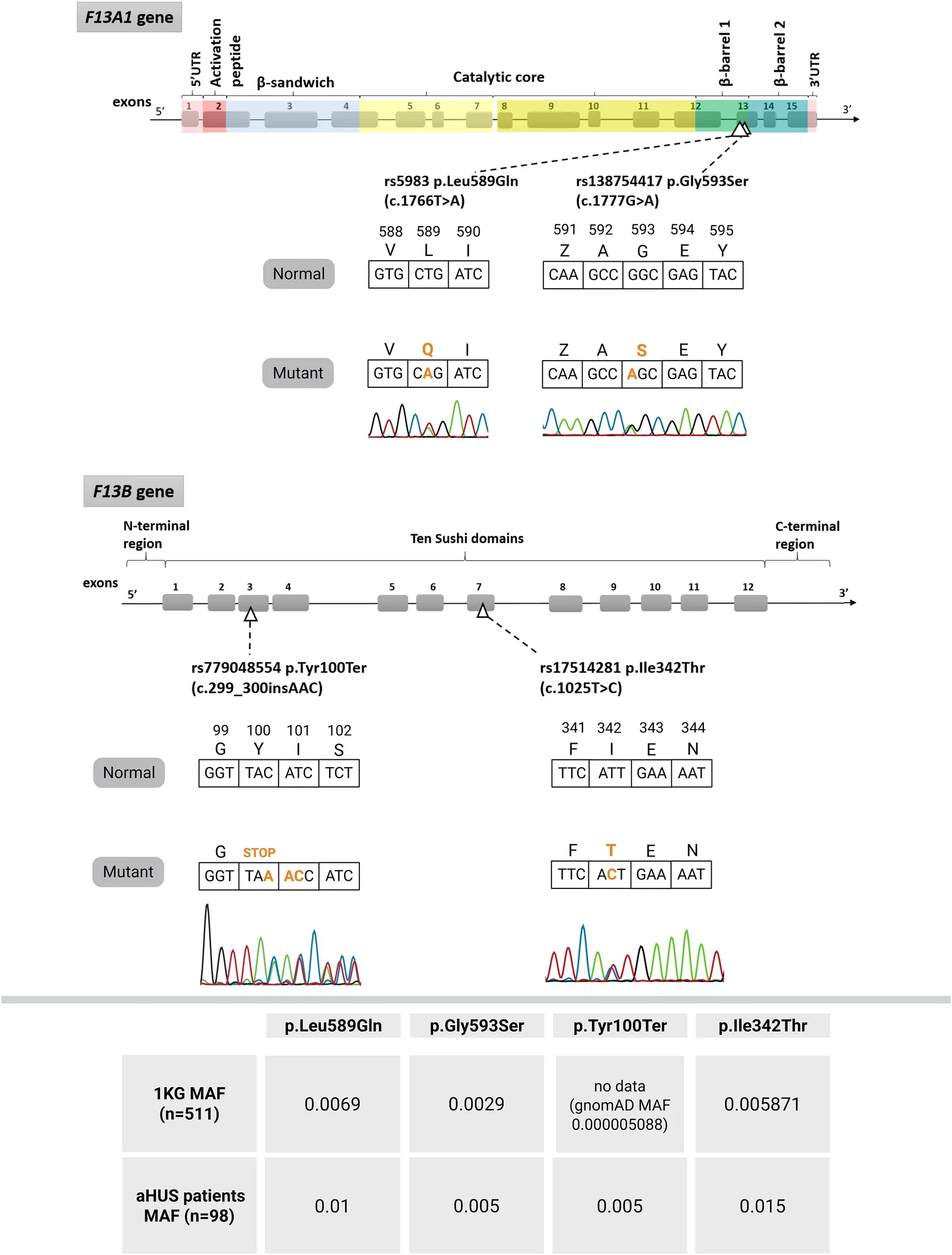
Detected rare variants in *F13A1* and *F13B* genes, and their minor allele frequencies (MAFs) in our aHUS cohort and in the European subset of the 1000Genomes (1KG) Project. aHUS, atypical hemolytic uremic syndrome; UTR, untranslated region. Created in BioRender.com.

By screening the *F13A1* and *F13B* genes, 2 rare missense *F13A1* variants (Leu589Glu rs5983, Gly593Ser rs138754417) and 2 rare nonsynonymous *F13B* variants (Ile342Thr rs17514281, Tyr100Ter rs779048554) were identified in aHUS patients, all in the heterozygous state. The detected genetic alterations with a minor allele frequency <0.01 in the European healthy population from the 1000Genomes Project were defined as rare variants. The location of the identified rare variants and their minor allele frequency in aHUS patients as well as in healthy individuals is shown in Figure 3. No association was found between the rare variants and aHUS using enrichment analysis.

The Tyr100Ter variant was not identified in healthy populations in the 1000Genomes Project; therefore, allele frequency reported for the non-Finnish European population in the Genome Aggregation Database (gnomAD) is provided.

**Figure 4:**
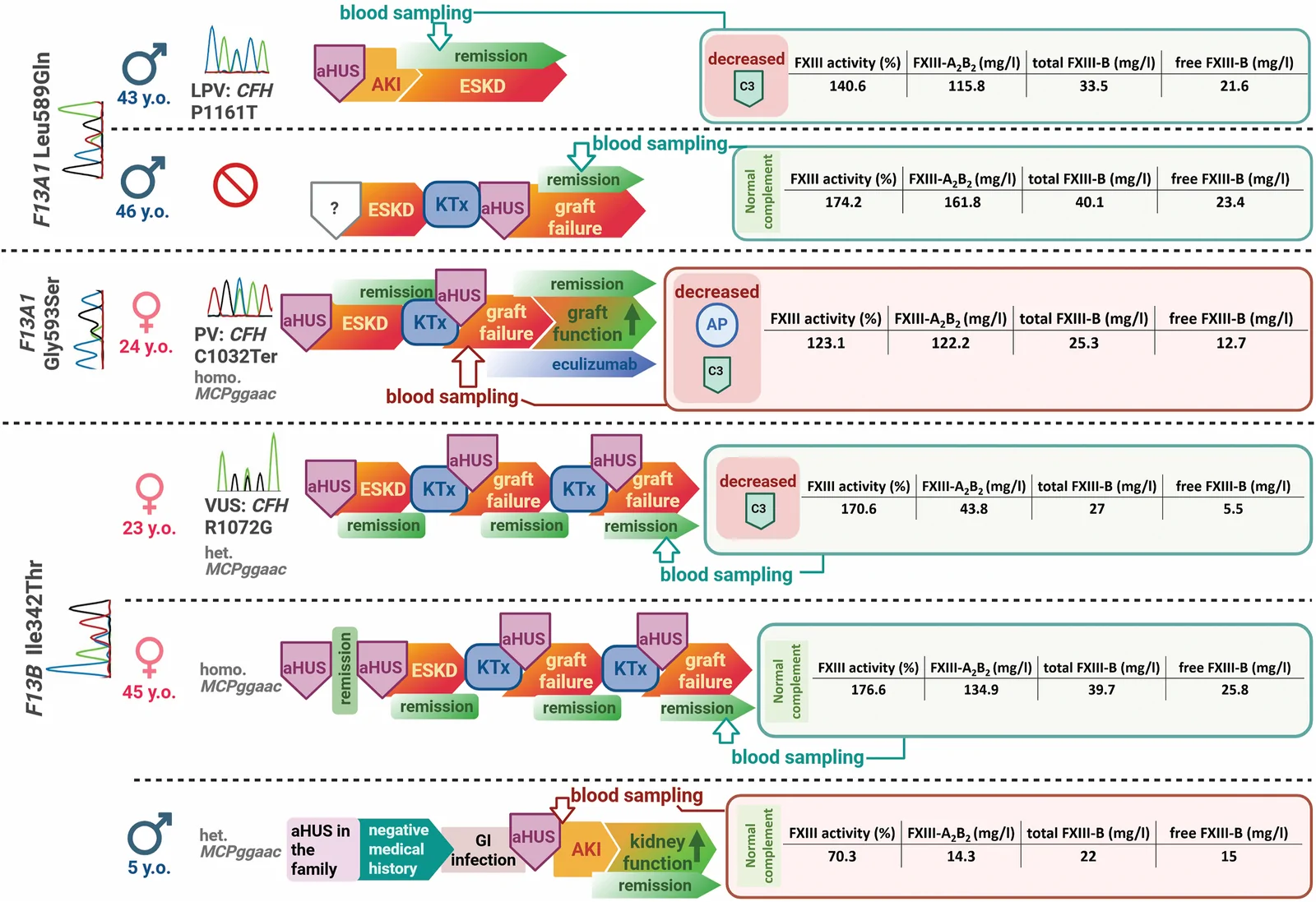
Summary of clinical data, complement abnormalities, and known aHUS-associated variants in patients carrying rare missense variants in *F13A1* or *F13B.* The Leu589Gln *F13A1* rare variant was identified in heterozygous form in 2 unrelated patients within our aHUS cohort. One had a single episode of aHUS that ultimately led to ESKD. This case, previously reported by Szarvas et al. [21], also carried a pathogenic *CFH* variant, and slightly reduced C3 levels were found in remission. The second patient developed aHUS after kidney transplantation for an unknown primary renal disease, had no rare complement gene variants, and exhibited normal complement levels in remission. In both Leu589Gln carriers, plasma FXIII levels appeared unaltered. The missense variant Gly593Ser in the *F13A1* gene was identified in a young female patient who required kidney transplantation due to aHUS, a case that has been reported previously [22,23]. She also carries a pathogenic *CFH* variant (Cys1032Ter) in heterozygous form and the *MCPggaac* risk haplotype in homozygous state. During recurrent aHUS before eculizumab, AP and C3 were decreased in line with disease activity, whereas FXIII levels were unchanged. The Ile342Thr *F13B* variant was detected in heterozygous form in 3 unrelated aHUS patients. One young female had childhood-onset disease with multiple relapses and repeated graft losses after several kidney transplants, as published previously [24]. She also carries a rare *CFH* VUS and the *MCPggaac* haplotype in heterozygous form, with low C3 levels during follow-up. A second young female had 2 adult-onset aHUS episodes and 2 kidney transplants complicated by early TMA recurrence. She carried only the *MCPggaac* haplotype in homozygous form without rare complement variants. The third, a 5-year-old patient, experienced his first aHUS episode after a non-verotoxin GI infection, had a positive family history, and carried the *MCPggaac* haplotype in heterozygous state but no rare risk variants in the studied genes. The plasma FXIII levels of these 3 patients were heterogeneous, consistent with differences in disease activity rather than a shared FXIII-related genetic mechanism. aHUS, atypical hemolytic uremic syndrome; AKI, acute kidney injury; AP, alternative pathway activity; C3, complement C3; ESKD, end-stage kidney disease; FXIII, factor XIII; het., heterozygous; homo., homozygous; LPV, likely pathogenic variant; KTx, kidney transplantation, PV, pathogenic variant; remission, hematologic remission; VUS, variant of uncertain significance; y.o., years old. Created in BioRender.com.

**Figure 5:**
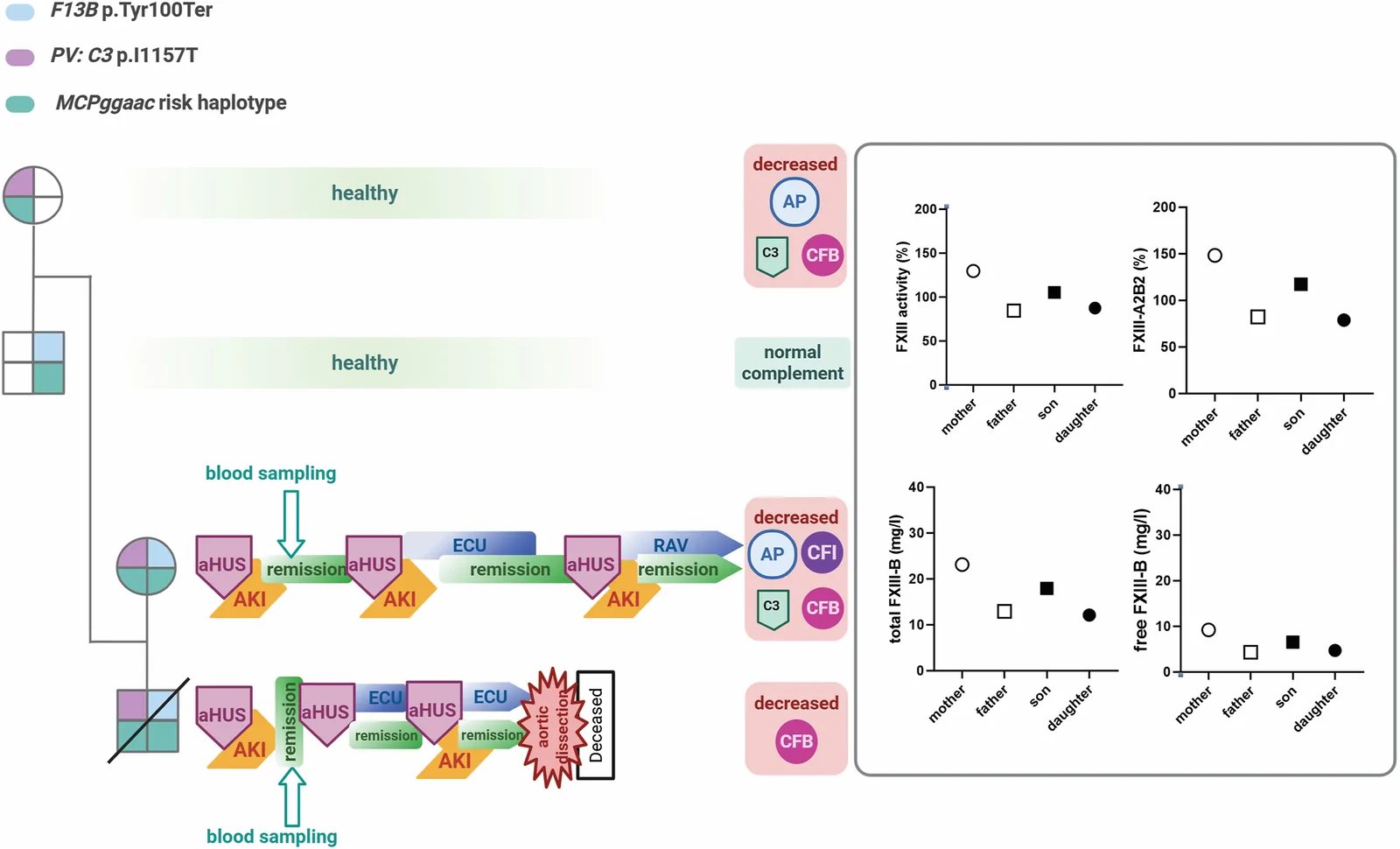
Pedigree of the family carrying the *F13B* Tyr100Ter variant, with corresponding serum complement and plasma FXIII levels and clinical history. The rare variant Tyr100Ter in the *F13B* gene was identified in heterozygous form in 2 siblings with aHUS, as well as in their healthy father. The 2 affected siblings were previously reported by Szarvas et al. [21]. Both affected children experienced multiple episodes of aHUS and responded to eculizumab therapy; however, aHUS recurred in both after discontinuation of complement inhibition. The daughter is currently receiving ravulizumab therapy, whereas the son unexpectedly died due to an aortic dissection. Previous analyses indicated that the affected siblings and their healthy mother also carry a rare pathogenic variant in the *C3* gene in heterozygous form. All family members under study bear the *MCPggaac* haplotype, with the parents exhibiting it in heterozygous form and the children in homozygous form. Although the children were in hematologic remission at the time of blood sampling, the analysis of their serum complement profile revealed signs of complement consumption. Notably, decreased levels of serum C3, CFB, and AP activity were observed in the healthy mother. The levels of FXIII activity, FXIII-A_2_B_2_, total and free FXIII-B in the family members exhibited a similar trend. These plasma parameters were found to be decreased in the *F13B* variant carrier family members. AKI, acute kidney injury; AP, alternative pathway activity; C3, complement C3; CFB, complement factor B; CFI, complement factor I; ECU, eculizumab, FXIII, factor XIII; PV, pathogenic variant; RAV, ravulizumab. Created in BioRender.com.

The clinical characteristics of aHUS patients carrying the detected rare *F13A1* and *F13B* variants are detailed in Figure 4 [[21], [22], [23], [24]] and Figure 5 [21].

The Tyr100Ter variant is an insertion of 3 bases (c.299_300insAAC) that results in the introduction of a premature stop codon. This variant was identified in heterozygous form in the *F13B* gene of a female adolescent patient with aHUS. Subsequent family screening revealed that her affected brother and their healthy father also carried the Tyr100Ter variant in heterozygous form. The genetic background of the family, along with serum complement alterations and plasma FXIII levels, are shown in Figure 5.

## 4 Discussion

This study aimed to determine whether aHUS is associated with characteristic alterations in FXIII plasma levels and to identify potential genetic risk variants in the *F13A1* and *F13B* genes in affected patients. Decreased FXIII activity and FXIII-A_2_B_2_ levels were detected in patients in the acute phase of aHUS, accompanied by increased concentrations of free FXIII-B compared with healthy controls. In clinical remission, FXIII activity and FXIII-A_2_B_2_ normalized, whereas both total and free FXIII-B levels increased further, reaching higher concentrations than those observed in controls and during the acute phase. No genetic basis for the observed plasma alterations was identified in the *F13A1* and *F13B* genes. Alleles of the common SNPs and rare variants were not enriched in patients with aHUS, and carriers of these variants did not appear to show any consistent clinical pattern over the course of the disease.

To our knowledge, this is the first study to provide a comprehensive assessment of both plasma FXIII levels and FXIII genetics in patients with aHUS.

Decreased FXIII activity and FXIII-A_2_B_2_ levels were observed in patients with acute aHUS compared with those in remission and healthy controls. FXIII plasma levels have previously been investigated in thrombotic disorders such as deep vein thrombosis [25], pulmonary embolism [26], myocardial infarction [27], and ischemic stroke [28]. In these conditions, reduced FXIII activity in the acute phase has been attributed to consumption and has been shown to normalize after the event [25,26]. These findings are consistent with our results.

Reduced FXIII activity and lower plasma levels of FXIII-A_2_B_2_ detected during the acute phase of aHUS in our cohort were accompanied by elevated concentrations of free FXIII-B. Research on the FXIII-B subunit has predominantly focused on its stabilizing interaction with FXIII-A and on its reduction in deficiency states [29]. Because the FXIII-B subunit is generally regarded as a carrier protein for FXIII-A rather than an independent functional unit with enzymatic activity [13], only a few studies have investigated plasma levels of free FXIII-B in thrombotic disorders. Elevated FXIII-B concentrations were reported in 2 stroke cohorts (one including patients with acute cerebral infarction and primary intracerebral hemorrhage, the other nonacute ischemic stroke) [30,31], whereas no changes in FXIII-B levels were detected in studies of acute myocardial infarction and acute pulmonary embolism [27,32].

It can be hypothesized that as FXIII-A becomes progressively incorporated into fibrin thrombi during the acute phase of aHUS [33], increasing dissociation of the FXIII-A_2_B_2_ complex releases free FXIII-B, explaining the higher free FXIII-B levels in the acute stage. However, in our study, FXIII-B concentrations were highest in remission despite normalization of FXIII activity and FXIII-A_2_B_2_ levels. This increase in total FXIII-B appears to be driven predominantly by expansion of the free FXIII-B fraction rather than changes in the FXIII-A_2_B_2_ complex. FXIII-B circulates in excess over FXIII-A and follows distinct plasma kinetics; hepatocytes produce FXIII-B at a high constitutive rate, and its plasma concentration is not solely dependent on the A subunit [34]. Because remission samples were obtained months to years after the acute episode, any transient FXIII-A_2_B_2_/free FXIII-B shift secondary to substantial FXIII-A consumption earlier in the disease course is unlikely to explain the persistently elevated free FXIII-B observed in remission. However, elevated D-dimer indicates ongoing lysis of cross-linked fibrin and therefore prior FXIII activation, suggesting some degree of FXIII-A consumption not reflected in FXIII-A_2_B_2_ or activity levels, which remain comparable to those in healthy controls [35].

Remission in our cohort was defined hematologically (absence of hemolysis and normalized platelet counts) without requiring renal recovery, and 35% of patients in remission had progressed to end-stage kidney disease (ESKD). Although renal impairment alters plasma protein kinetics, current evidence does not support renal clearance as a major elimination pathway for free FXIII-B [36]. Data on FXIII in chronic kidney disease and ESKD are very limited: Pénzes et al. [37] and Caruana et al. [38] reported elevated FXIII activity and FXIII-A_2_B_2_ levels together with increased predialysis fibrinogen concentrations in ESKD patients. In contrast, in our aHUS remission cohort, fibrinogen, FXIII activity, and FXIII-A_2_B_2_ levels were all comparable to those of healthy controls. To our knowledge, no studies have specifically evaluated free FXIII-B levels in this population.

Although free FXIII-B and fibrinogen were positively correlated in our cohort, fibrinogen itself was not elevated, indicating that increased free FXIII-B is not merely a surrogate of fibrinogen dynamics, nonspecific acute phase activation, or the procoagulant state characteristic of ESKD [39,40]. Rather, this pattern is consistent with pathophysiologically plausible, disease-specific processes. These include low-grade endothelial or complement activation with associated alterations in fibrinolytic balance. Such mechanisms may contribute to an expanded free FXIII-B pool beyond that attributable to FXIII-A consumption alone [41].

As FXIII levels may be influenced by variants in the genes encoding the A and B subunits of FXIII, we performed HRM-based screening, followed by Sanger sequencing validation, to identify variants in the coding regions of these genes. Until now, numerous studies have assessed the genetic background of aHUS using next-generation sequencing methods; however, in most cases, a panel-based technique was applied focusing only on aHUS-related genes. Nevertheless, a few studies applied a nontargeted approach when analyzing whole exome sequencing, but even these unbiased investigations reported no genetic alterations in genes encoding FXIII [6,[40], [41], [42]].

In our study, 6 common and 4 rare nonsynonymous variants were identified in the *F13A1* and *F13B* genes, all of which were previously reported in databases. It should be noted, however, that our HRM-based approach targets the coding regions and splice sites only; deep intronic or structural variants would not be captured by this design.

One of the common SNPs, Val35Leu in *F13A1*, has previously been examined in TMAs. Homozygosity for Leu35 was associated with an increased risk of thrombotic thrombocytopenic purpura-HUS in a small cohort (*n* = 40) [12], but in our larger aHUS cohort, allele and genotype frequencies were comparable to those of healthy controls, and we could not confirm this association. Similarly, no differences were observed between aHUS patients and controls in the allele or genotype frequencies of the 5 other common variants.

Among the 4 identified rare nonsynonymous variants, one was extremely rare in healthy populations (detected in 6 of 589,631 individuals in the gnomAD non-Finnish European cohort and absent from the 1000Genomes database) and was found in 1 of the 98 aHUS patients and in his sibling, who was also affected by aHUS. This variant (Tyr100Ter in *F13B*); was previously identified in homozygous form in 2 patients with type I FXIII deficiency [42], leading to absent or nonsecreted truncated protein and secondary FXIII-A deficiency. In our cohort, carriers exhibited reduced FXIII activity, FXIII-A_2_B_2_, and free FXIII-B levels, consistent with partial FXIII-B deficiency, although a pathogenic contribution of this *F13B* variant to the disease course remains uncertain.

The other 3 rare variants identified in this study (Leu589Gln and Gly593Ser in the *F13A1* gene and the Ile342Thr in the *F13B* gene) were found in aHUS patients at frequencies comparable to those reported in healthy populations. It should be noted that the relatively small cohort size limits the interpretability of these frequency estimates, and replication of the genetic analysis in a larger cohort will be required to confirm these findings. These 3 rare variants were previously considered variants of uncertain functional significance, with no apparent influence on FXIII plasma parameters [[43], [44], [45], [46], [47]]. Evaluation of the clinical trajectory in the 9 patients carrying *F13A1* or *F13B* rare variants—including Tyr100Ter carrier family members—revealed no discernible pattern regarding trigger events, overall episode courses, or outcomes. However, this conclusion is limited by the retrospective, observational, nonrandomized design and small cohort size.

Taken together, this study demonstrates for the first time that FXIII-B levels are elevated during remission of aHUS, representing a novel feature that suggests altered FXIII-B kinetics potentially driven by persistent endothelial or complement activation, altered hepatic synthesis, or modified clearance. A key limitation of this study is that individual hematologic parameters at the time of sampling were not available in our laboratory dataset. Moreover, only 4 patients contributed both acute phase and remission samples; for all other participants, comparisons between disease phases and with controls are cross-sectional, which limits inferences about within-patient temporal dynamics. Therefore, future prospective studies should include contemporaneous collection of plasma variables. This would allow direct assessment of associations between FXIII levels and complement activity, hemolysis, platelet count, renal function, and clinical relapses. Such studies should also define the dynamics of free FXIII plasma levels across acute, remission, and long-term phases. Systematic assessment may help clarify the relevance of free FXIII-B as a marker of subclinical disease activity. As fibrin-rich thrombosis is a shared hallmark of TMAs, extending these investigations to other hereditary and acquired forms will be essential to assess the generalizability of these findings. In this context, identifying subclinical disease activity is critical for management. Detecting complement-mediated endothelial injury before overt thrombocytopenia or hemolysis could prompt closer monitoring or earlier initiation of complement inhibition to prevent irreversible renal damage. Conversely, confirming disease quiescence may support safe discontinuation of complement inhibitors [48].

In conclusion, this first comprehensive genetic and plasma analysis of FXIII in aHUS demonstrates that the absence of rare variant enrichment, and persistent FXIII deficiency in remission argues against a causal role of FXIII in disease pathogenesis. However, the remission-associated increase in FXIII-B, whether it reflects an underlying predisposing factor or a consequence of the preceding microangiopathic event, highlights this protein as a promising biomarker candidate and opens a clinically meaningful direction for future research, requiring further validation.

## Figures and Tables

**Supplementary Figure:**